# Conversion of C_6_ and C_5_ sugars in undetoxified wet exploded bagasse hydrolysates using *Scheffersomyces* (*Pichia) stipitis* CBS6054

**DOI:** 10.1186/2191-0855-3-42

**Published:** 2013-07-29

**Authors:** Rajib Biswas, Hinrich Uellendahl, Birgitte K Ahring

**Affiliations:** 1 Section for Sustainable Biotechnology, Aalborg University Copenhagen, Copenhagen, Denmark; 2 Center for Bioproducts and Bioenergy, Washington State University, 2710 University Drive, Richland, WA 99354-1671, USA

**Keywords:** *Scheffersomyces (Pichia) stipitis*, Cellulosic ethanol, Sugarcane bagasse, Wet explosion pretreatment, Inhibitors, Xylose fermentation

## Abstract

Sugarcane bagasse is a potential feedstock for cellulosic ethanol production, rich in both glucan and xylan. This stresses the importance of utilizing both C_6_ and C_5_ sugars for conversion into ethanol in order to improve the process economics. During processing of the hydrolysate degradation products such as acetate, 5-hydroxymethylfurfural (HMF) and furfural are formed, which are known to inhibit microbial growth at higher concentrations. In the current study, conversion of both glucose and xylose sugars into ethanol in wet exploded bagasse hydrolysates was investigated without detoxification using *Scheffersomyces (Pichia) stipitis* CBS6054, a native xylose utilizing yeast strain. The sugar utilization ratio and ethanol yield (Y_p/s_) ranged from 88-100% and 0.33-0.41 ± 0.02 g/g, respectively, in all the hydrolysates tested. Hydrolysate after wet explosion at 185°C and 6 bar O_2_, composed of mixed sugars (glucose and xylose) and inhibitors such as acetate, HMF and furfural at concentrations of 3.2 ± 0.1, 0.4 and 0.5 g/l, respectively, exhibited highest cell growth rate of 0.079 g/l/h and an ethanol yield of 0.39 ± 0.02 g/g sugar converted. *Scheffersomyces stipitis* exhibited prolonged fermentation time on bagasse hydrolysate after wet explosion at 200°C and 6 bar O_2_ where the inhibitors concentration was further increased. Nonetheless, ethanol was produced up to 18.7 ± 1.1 g/l resulting in a yield of 0.38 ± 0.02 g/g after 82 h of fermentation.

## Introduction

In recent years, ethanol production from renewable sources has received increased attention in a world of dwindling fossil fuels reserves along with the environmental concerns. Commercial production of bioethanol is mostly driven by starch- or sucrose-containing feedstocks such as corn, sugarcane, wheat by fermentation with *Saccharomyces cerevisiae* (Wheals et al. 1999). Non-food feedstocks, however, such as lignocellulosic materials including agricultural wastes such as bagasse hold significant potential and have been identified as suitable feedstock sources for ethanol production (Lynd et al. 1991). Lignocellulose based ethanol processes require pretreatment as a first step followed by enzymatic hydrolysis of carbohydrates (Ahring et al. 1996; Margeot et al. 2009). Unlike the hydrolysis of starch- and sugar-based feedstock that results primarily in hexoses, lignocellulose is composed of cellulose and hemicellulose, resulting in both hexoses (C_6_) and pentoses (C_5_) sugars (Rubin 2008). An efficient pretreatment strategy along with the fermentation of C_6_ and C_5_ sugars are keys to bring cellulosic ethanol to commercial reality.

Sugarcane bagasse (SCB), the residual plant material of sugarcane, is one of the most abundant lignocellulosic feedstocks suitable for ethanol production (Cardona et al. 2010; Pandey et al. 2000). In addition, its on-site availability at sugarcane-based ethanol process plants is advantageous for large-scale processing. Currently the bagasse generated after sucrose extraction from sugarcane is incinerated to power the plant operation (Shi et al. 2012). SCB is primarily composed of cellulose (40-45%), hemicelluloses (30-35%) and lignin (20-30%) (Cardona et al. 2010). Cellulose is a D-glucose polymer while hemicellulose predominantly consists of D-xylose, a five-carbon sugar (Girio et al. 2010; Jeffries et al. 2007; Skoog and Hahn-Hägerdal 1990). An appropriate pretreatment is essential for efficient enzymatic saccharification (Ahring et al. 1996). Various pretreatment methods have shown the potential to disrupt the cell wall structure of SCB to facilitate the enzymatic hydrolysis of the polysaccharides (Cardona et al. 2010; Martin et al. 2007). Wet explosion is a thermochemical pretreatment method, where biomass is treated at high temperature and pressure. Typically an oxidizing agent such as elemental oxygen or H_2_O_2_ is added to help disrupt the cell wall structure, and solubilize hemicellulose and lignin. The process is terminated by sudden pressure release to a subsequent flash tank (Ahring and Munck 2006; Rana et al. 2012). In previous studies, the potential of wet explosion pretreatment of bagasse to facilitate saccharification at low enzyme dosage was demonstrated (Biswas et al. unpublished). The oxidative pretreatment strategy was found to improve the cellulose conversion to glucose in the subsequent enzymatic hydrolysis, as well as producing high xylose yields through solubilization of hemicellulose. However, during the processing of hydrolysate for subsequent microbial fermentation, degradation products such as acetate, 5-hydroxymethylfurfural (HMF), furfural will be formed to various degree known to inhibit the microbial growth and product yields at higher concentration (Bellido et al. 2011; Nigam 2001a; Palmqvist and Hahn-Hägerdal 2000).

The importance of utilizing all hydrolyzed sugar monomers into ethanol for improving process economics is self-evident. *Saccharomyces cerevisiae* is the most commonly used yeast for industrial ethanol fermentation, only capable of glucose fermentation. Some naturally occurring yeast such as *Scheffersomyces stipitis*, *Candida shehatae*, and *Pachysolen tannophilus* are able to ferment both hexoses and pentoses to ethanol. Among the xylose fermenting yeasts, *Scheffersomyces stipitis* seems to be the most promising strain for industrial application due to its high ethanol yield. In addition, this organism is able to ferment most of the sugars glucose, xylose, mannose, galactose and cellobiose (Agbogbo and Coward-Kelly 2008). However, previous studies have shown arabinose is only utilized by *S. stipitis* for cell growth but not for ethanol production (Nigam 2001b). Furthermore, *S. stipitis* also has the natural ability to metabolize some of the sugar degradation compounds present in the hydrolysate after pretreatment (Almeida et al. 2008; Wan et al. 2012). The sensitivity of *Scheffersomyces stipitis* to inhibitors found in lignocellulose hydrolysate has been reported elsewhere (Bellido et al. 2011; Delgenes et al. 1996).

Inhibitory compounds, such as acetic acid, HMF and furfural are produced in different concentrations depending on the pretreatment severity and can inhibit the growth of yeast cell and thus lower the yield and productivity of ethanol fermentation. It was previously reported that prolonged incubation helps to acclimatize *Scheffersomyces stipitis* to these toxic compounds (Delgenes et al. 1996). In the present study, we investigated conversion of both hexose and pentose sugars in the enzymatic hydrolysates of wet exploded sugarcane bagasse without detoxification of the inhibitors to study cell growth and ethanol yields by *S. stipitis* CBS6054. We further compared the cell growth and yields using bagasse xylose hydrolysate containing only xylose with lower concentrations of the inhibitors. The kinetics of cell growth in the hydrolysates compared to synthetic media was also assessed.

## Materials and methods

### Yeast strain and inoculum preparation

*S. stipitis* CBS6054 was obtained from the American Type Culture Collection (ATCC) and was preserved at −80°C in the Bioproducts, Sciences and Engineering Laboratory (BSEL), Washington State University (WSU), USA. The organism was cultivated in a media previously described elsewhere (Agbogbo and Wenger 2006, 2007). A mixture of yeast extract, urea, peptone and xylose (YUPX) in the respective proportions of 1.7, 2.27, 6.65 and 20.0 g/l was filter sterilized (0.22 μm) and used as source of nutrient. 250 ml sterilized Erlenmeyer baffled flasks were used and inoculation was done aseptically. The inoculated medium was incubated in a shake incubator (The Lab Companion IS-971 (R/RF) Floor Model Incubated Shaker, GMI Inc., USA) at 30°C and agitation speed of 140 rpm for 48 h. Microaerobic conditions were maintained by using foam plugs on the Erlenmeyer flasks (Identi-Plugs®, Jaece Industries, Inc., NY). *S. stipitis* cells were harvested towards the end of the exponential growth phase by centrifugation at relative centrifugal force (RCF) 3824 × g for 10 minutes. The harvested cells were washed twice and resuspended in sterilized distilled water in the desired cell concentration and served as inoculum.

### Wet explosion pretreatment

Wet explosion pretreatment was performed using the WSU pretreatment pilot plant for disrupting the lignocellulosic matrix and fractioning the lignin and hemicellulosic components as previously described (Rana et al. 2012). Sugarcane bagasse was added to the 10 l pretreatment reactor as wet slurry with 16% dry matter concentration, containing 640 g of oven dried bagasse and 3343 g of tap water. The reactor was hermetically closed, 6 bar of O_2_ was then purged into the reactor with the headspace of 6 l and the reactor was heated to the desired temperature. Reaction time was 10 minutes at the desired temperature and pressure. Three suitable pretreatment conditions were chosen based on preliminary results on enzymatic hydrolysis of wet exploded bagasse (Table 1). Higher enzyme efficiency and recovery of both glucose and xylose were obtained under condition B followed by condition C, while condition A was found suitable for especially xylose recovery and formation of inhibitors such as weak acid is minimal. Therefore, condition A was chosen for a control condition to obtain hydrolysate contained mostly xylose.

**Table 1:** Wet explosion pretreatment conditions applied on sugarcane bagasse with a treatment time of 10 minutes

**Pretreatment**	** Temperature,**	**O**_**2 **_	**pH**		**Dry matter,%**	
	**°C**	**used**	** Initial**	** Final**	** Initial**	** Final**
		**(bar)**				
A	170	6	5.85	3.12	16.0	15.5
B	185	6	5.85	3.05	16.0	16.2
C	200	6	5.85	2.93	16.0	14.0

### Preparation of hydrolysate from wet exploded bagasse

#### Xylose hydrolysate after SSF

A liquid fraction (A_X_) containing mostly xylose as fermentable sugar was obtained after simultaneous saccharification and fermentation (SSF) of wet exploded bagasse at condition A (Tables 1 and 2). *Saccharomyces cerevisiae* was used for removing the fermentable glucose for an incubation period of 162 hours. Same enzyme loading of 12.4 mg enzyme protein (EP)/g cellulose at 10.1 ± 0.1% dry matter was used for the SSF. Since only glucose is utilized by the strain, the remaining liquid fraction after the SSF contained mostly xylose as fermentable sugar. After the fermentation was completed, ethanol produced during SSF was removed by vacuum distillation and the liquid fraction rich in xylose (A_X_) was separated for further use.

**Table 2:** **Composition (g/l) of the substrates used for fermentation by *Scheffersomyces (Pichia) stipitis***

**Substrate**	**Initial sugar concentration**		**Initial inhibitor concentration**		
	**Glucose**	**Xylose**	**Acetic acid**	**HMF**	**Furfural**
A_X_ ^*a*^	0.0	14.7 ± 0.0	1.0 ± 0.0	1.2 ± 0.0	0.4 ± 0.0
B_GX_ ^*b*^	17.3 ± 0.5	9.6 ± 0.2	3.2 ± 0.1	0.4 ± 0.0	0.5 ± 0.0
C_GX_ ^*c*^	42.8 ± 0.8	6.3 ± 0.0	6.9 ± 0.1	1.2 ± 0.0	0.8 ± 0.0
S_GX_ ^*d*^	6.1 ± 0.0	15.2 ± 0.0	0.0	0.0	0.0
S_G_ ^*d*^	27.2 ± 0.5	0.0	0.0	0.0	0.0
S_X_ ^*d*^	0.0	25.6 ± 0.4	0.0	0.0	0.0

^*a*^ hydrolysate after pretreatment at condition A and the simultaneous saccharification and fermentation (SSF) using *Saccharomyces cerevisiae*.

^*b*^ hydrolysate after pretreatment at condition B and enzymatic hydrolysis.

^*c*^ hydrolysate after pretreatment at condition C and enzymatic hydrolysis.

^*d*^ respective commercial sugar (granular powder) was used as control substrate, Fisher Chemical, USA.

#### Hydrolysate with mixed sugars after enzymatic hydrolysis

After pretreatments under condition B and C (Table 1), enzymatic hydrolysis was carried out on the whole wet exploded material (slurry) without any solid–liquid separation. For saccharification, a mixture of the two commercial enzymes Cellic® CTec2 and Cellic® HTec2 (Novozymes, USA) were used in a ratio of 85:15 (%, v/v), respectively, with the enzyme loading of 12.4 enzyme protein (EP)/g cellulose at 10.1 ± 0.1% dry matter. The enzyme protein (EP) content of Cellic® CTec2 and Cellic® HTec2 determined prior to enzymatic hydrolysis were 279 ± 8 and 251 ± 12 mg EP/ml, respectively. Enzymatic hydrolysate B_GX_ and C_GX_ were obtained from enzymatic hydrolysis of the pretreated samples under condition B and C, respectively (Table 2). Hydrolysates were always filter sterilized (0.2 μm, Millipore, USA) prior to inoculation.

##### Shake flask fermentation

Shake flask fermentation was conducted in duplicates with the hydrolysates (Table 2) under same conditions as previously described. Filter sterilized synthetic medium S_GX_, S_G_ and S_X_ were prepared using commercial sugar(s) (Fisher Chemical, USA) contained glucose + xylose, glucose and xylose, respectively, with the concentration as depicted in Table 2. Erlenmeyer baffled flasks were used with a volume of 50 ml. Adjustment of pH to 6.0 ± 0.5 was performed for hydrolysates with 1 M NaOH whenever this was needed to ensure a pH of at least 6.0. Each flask contained 30 ml of hydrolysate or sugars solution (glucose and/or xylose in DI water), 1 ml of nutrient solution and 1 ml of inoculum (initial cell concentration 1 g/l). Nutrient solution was prepared by dissolving 4.25 g of yeast extract, 5.68 g of urea and 16.40 g of peptone in 23.68 ml of water to reach a volume of 50 ml. All fermentation flasks were supplemented with sufficient carbon sources (i.e., hydrolysate or commercial sugar) and nutrients to produce equivalent amount of cell mass and to exhibit similar growth rates under the favorable conditions ensured. The flasks were incubated for 106 hours except for hydrolysate C_GX_ which was incubated for 174 hours. 2 ml of sample was withdrawn after 0, 6, 12, 24, 36, 48, 58, 82, 106 and 174 hours (in case of hydrolysate C_GX_) for analysis of sugar and inhibitor concentrations, cell concentration and pH.

##### Analytical methods

Cell concentrations were determined by optical density (OD) measurement of the cells using spectrophotometer (Jenway 6405 UV/Visible, NJ, USA) system at 600 nm (1 OD = 0.17 g/l of dry cells). Glucose, xylose, arabinose, acetic acid, ethanol, HMF and furfural were quantified by HPLC on an Aminex HPX-87H column (Bio-Rad, Hercules, USA) at 60°C with 4 mM H_2_SO_4_ as an eluent with a flow rate of 0.6 ml/min. HPLC was equipped with refractive index and UV visible detector. All samples were filtered through a 0.45 μm PTFE membrane (Acrodisc® Syringe Filters, 13 mm, Pall® Life Sciences, USA) prior to HPLC analysis. The pH was monitored using InLab® Micro combination pH electrode (precision ± 0.001 pH).

## Results

### Effects of inhibitors on sugar utilization and ethanol yields

**Figure 1:**
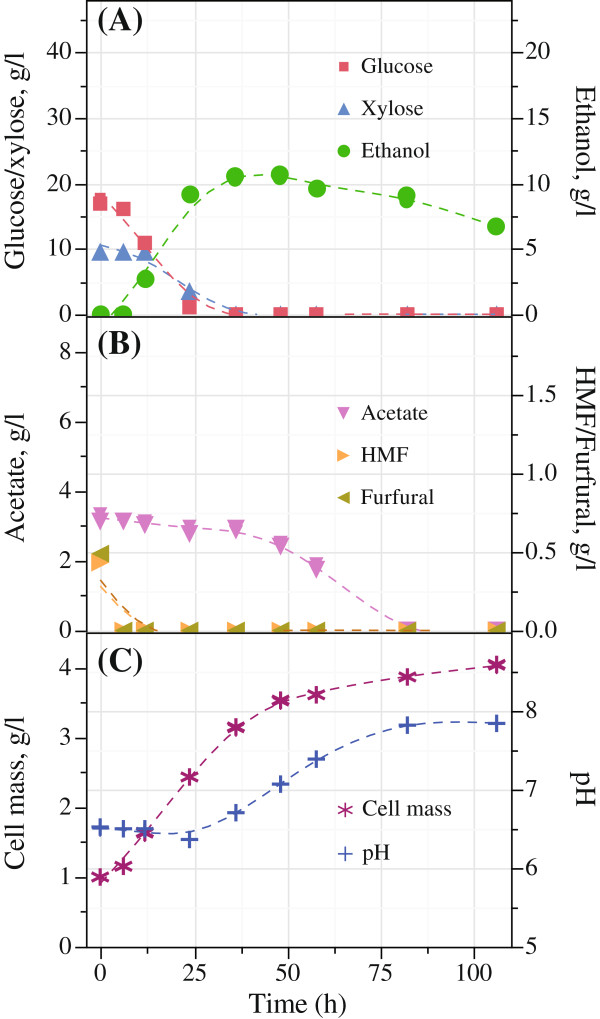
**Fermentation results for hydrolysate B**_**GX **_**obtained after enzymatic hydrolysis of wet exploded bagasse under condition at 185°C with 6 bar O**_**2**_**; (A) sugar conversion and ethanol production; (B) conversion of inhibitors; and (C) cell growth and pH in batch fermentation with *Scheffersomyces (Pichia) stipitis *CBS6054.**

**Figure 2:**
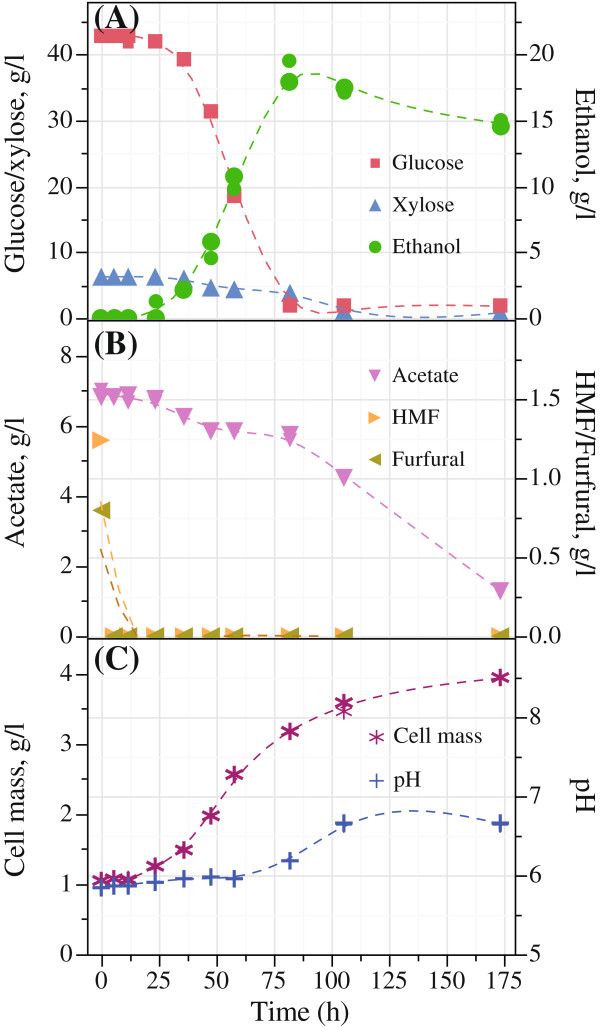
**Fermentation results for hydrolysate C**_**GX **_**obtained after enzymatic hydrolysis of wet exploded bagasse under condition at 200°C with 6 bar O**_**2**_**; (A) Sugar conversion and ethanol production; (B) conversion of inhibitors; and (C) cell growth and pH in batch fermentation with *Scheffersomyces (Pichia) stipitis *CBS6054.**

The main parameters measured for the fermentation by *Scheffersomyces stipitis* CBS6054 on the different hydrolysates and control media are displayed in Table 3. Sugar utilization, ethanol yield, inhibitor concentration, pH and growth kinetics of hydrolysates B_GX_ and C_GX_ are presented in Figures 1 and 2, respectively.

**Table 3:** **Summaries of fermentation results at highest ethanol concentration time points using *Scheffersomyces (Pichia) stipitis***

**Substrate**	**Fermentation time,**	**Ethanol,**	**Sugar utilized,**	**Y**_p/s_^*^, g/g	**Cell growth rate,**	**Cell mass,**	**g/l at 106 h**	**pH at (f)h**
	**(f)h**	**g/l**	**%**		**g/l/h**	**g/l at (f)h**		
A_X_	58	6.1 ± 0.0	100 ± 0.0	0.41 ± 0.02	0.043	2.81 ± 0.02	3.34 ± 0.02	5.5 ± 0.0
B_GX_	36	10.4 ± 0.2	100 ± 0.0	0.39 ± 0.02	0.079	3.31 ± 0.00	4.02 ± 0.02	6.7 ± 0.0
C_GX_	82	18.7 ± 1.1	88 ± 0.0	0.38 ± 0.02	0.049	3.16 ± 0.00	3.52 ± 0.09	6.2 ± 0.0
S_GX_	76	8.2 ± 0.0	100 ± 0.0	0.39 ± 0.00	0.045	2.48 ± 0.00	2.69 ± 0.00	6.6 ± 0.0
S_G_	36	10.1 ± 0.1	99 ± 0.8	0.37 ± 0.00	0.064	2.33 ± 0.05	2.72 ± 0.04	6.0 ± 0.3
S_X_	82	8.5 ± 0.2	100 ± 0.0	0.33 ± 0.01	0.051	2.81 ± 0.03	2.94 ± 0.05	5.3 ± 0.1

^*^ Yp/s = ethanol yield coefficient, was calculated as the grams of ethanol produced per grams of sugar converted.

The sugar utilization ratio and ethanol yield (Y_p/s_) ranged from 88–100% and 0.33–0.41 ± 0.02 g/g, respectively, in all the hydrolysates and controls tested. The ethanol yields (Y_p/s_) of hydrolysate A_X_, B_GX_ and C_GX_ were 0.41 ± 0.02, 0.39 ± 0.02 and 0.38 ± 0.02 g/g, respectively. Ethanol yields were higher when using hydrolysates after pretreatment than control substrates, i.e., commercial sugars (Table 3).

The fermentation of xylose alone, after pretreatment at condition A (170°C, 6 bar O_2_) and SSF, took 58 h to convert 100% sugar (Table 3), which is longer than that of mixed sugars in the hydrolysate B_GX_ after pretreatment condition B (185°C, 6 bar O_2_), which took 36 h (Figure 1A). Both glucose and xylose were converted for the hydrolysates B_GX_ and C_GX_ obtained after the pretreatment and enzymatic hydrolysis of SCB, containing inhibitors in comparatively higher concentrations among others. Fermentation of enzymatic hydrolysate C_GX_ after pretreatment at condition C (200°C, 6 bar O_2_) resulted in a prolonged fermentation time of 82 h with initial lag phase of 12 h (Figure 2A). The delay in sugar conversion is likely due to the presence of inhibitors such as acetate, HMF and furfural at the concentrations of 6.9 ± 0.1, 1.2 and 0.8 g/l, respectively. Nonetheless, ethanol concentration was found to be 18.7 ± 1.1 g/l after 82 h of incubation. While *S. stipitis* adapted to the inhibitors, the fermentation was completed with an ethanol yield of 0.38 ± 0.02 g/g at 82 h. Although the utilization of sugars was limited to 88% within this time, sugar conversion was found to be 95% after 174 h of fermentation.

Taking into consideration that no detoxification was performed except the adjustment of pH with NaOH to 6.0 ± 0.5, it was found that the fermentation was only inhibited in bagasse hydrolysate C_GX_ after pretreatment at condition C (200°C, 6 bar O_2_). Acetic acid was converted in all fermentation experiments especially with hydrolysate B_GX_ and C_GX_ resulting an increase in pH (Agbogbo and Wenger 2007). After 82 h of fermentation, 100% acetic acid was metabolized in hydrolysate B_GX_ (Figure 1B). Hence, for the hydrolysate C_GX_, it took 174 h to bring the acetic acid concentration to 1.3 g/l from 6.9 ± 0.1 g/l (Figure 2B). Moreover, both HMF and furfural were utilized by *S. stipitis* CBS6054 within the first 12 hours of fermentation for hydrolysate B_GX_ and C_GX_.

### Effects of inhibitors on cell growth

When comparing the growth kinetics of *Scheffersomyces stipitis* CBS6054 in Figures 1C and 2C, the initial cell concentration of 1 g/l increased for all hydrolysates and grew to various final cell concentrations on the different hydrolysate medium. The highest amount of cell mass (g/l) produced in mixed sugars hydrolysate B_GX_ after 106 h of incubation was 4.02 ± 0.02, while 3.34 ± 0.02 and 3.52 ± 0.09 in hydrolysate A_X_ and hydrolysate C_GX_, respectively (Table 3).

Cell mass production was higher in all hydrolysates than found in synthetic medium (S_GX_, S_G_ and S_X_). Exponential growth was observed for hydrolysate A_X_ and B_GX_ (Figure 1C) during the initial 48 h without any noticeable lag phase. Cell mass in hydrolysate A_X_ and B_GX_ after 48 h were measured to 2.81 and 3.52 g/l, respectively. On the other hand, no cell growth was observed in hydrolysate C_GX_ within the first 12 h (Figure 2C).

The highest cell growth rate of 0.079 g/l/h was found in hydrolysate B_GX_ followed by 0.064 g/l/h in synthetic media S_G_ (Table 3). Acetic acid concentrations in the hydrolysates A_X_, B_GX_ and C_GX_ were 1.0 ± 0.0, 3.2 ± 0.1 and 6.9 ± 0.1 g/l, respectively (Table 2).

## Discussion

To realize the industrial ethanol production from hydrolysis of pretreated lignocellulose, it is essential to obtain strains capable of converting all the major sugars as well as being able to cope with the inhibitors present as sugar degradation product in the hydrolysate. Our present work demonstrates that the native strain *Scheffersomyces (Pichia) stipitis* CBS6054 is suitable for ethanol fermentation of both glucose and xylose present in hydrolysates of wet exploded bagasse without the need for detoxification, achieving substantial ethanol yields. The ethanol yield from xylose in the hydrolysate after pretreatment at 170°C with 6 bar O_2_ and SSF was 0.41 ± 0.02 g/g while a yield of 0.39 ± 0.02 g/g was achieved for the fermentation of glucose and xylose in the hydrolysate after pretreatment at 185°C with 6 bar O_2_ and enzymatic hydrolysis of wet exploded bagasse. The yields are in agreement with the results found in corn stover hemicellulose hydrolysate with similar inhibitor concentrations using *Scheffersomyces (Pichia) stipitis* CBS6054 (Agbogbo and Wenger 2007). Our results are comparable to those observed with adapted *S. stipitis* strains (Nigam 2001a,b). The utilization of glucose was more rapid than for xylose in the different hydrolysates. This similar observation in assimilation of sugars has been reported elsewhere (Agbogbo and Wenger 2007; Bellido et al. 2011; Nigam 2001a). In the presence of both glucose and xylose (B_GX_, C_GX_), conversion of glucose started prior to xylose conversion. In mixed substrate fermentation, significant xylose utilization is initiated by *Scheffersomyces (Pichia) stipites* once glucose concentration in the medium is below 20 g/l (Agbogbo et al. 2006).

Conversion of glucose and xylose was not completely inhibited for the hydrolysates B_GX_ and C_GX_, in the presence of known inhibitors such as acetate, HMF and furfural. Our study shows that the favorable growth condition for cell mass production is likely due to the mixed sugars, where glucose is converted more readily than xylose. Our results compare favorably with previous reports on fermentation of sugarcane bagasse hydrolysate (Rudolf et al. 2008). In contrast, Bellido et al. (2011) found that xylose was not utilized in 168 h of fermentation experiments using *Scheffersomyces (Pichia) stipitis* DSM3651 on filtered hydrolysate of steam exploded wheat straw using the whole slurry with acetate, HMF and furfural concentrations at 1.52, 0.05 and 0.14 g/l, respectively. Acetic acid is released from the esterified form of arabinoxylans during the processing of lignocellulose hydrolysate. The cleavage of the acetyl group occurs when lignocellulose undergoes high temperature, oxidation treatment and even in enzymatic hydrolysis process we further see a liberation of acetic acid. Previous studies showed the yeast cell growth is inhibited at an acetic acid concentration of about 2–5 g/l (Bellido et al. 2011; Nigam 2001a). Acetic acid is a weak acid having high pKa value of 4.75 (25°C) at zero ionic strength. pKa value refers to the pH value at which buffering capacity of the acid is highest and the concentration of dissociated and undissociated form of the acid are equal (Palmqvist and Hahn-Hägerdal 2000). The risk of inhibition due to liposoluble diffusion of undissociated weak acid across the plasma membrane can be reduced by increasing the pH (Palmqvist and Hahn-Hägerdal 2000). Therefore, favorable pH for the fermentation of the hydrolysates containing acetic acid will be between 5.5 and 6.5. Our study suggests that acetic acid can be utilized by *S. stipitis* as a substrate at a lower concentration that may not be inhibitory for cell growth at starting pH between 6.0 and 6.5. A similar observation of acetic acid conversion by *Scheffersomyces (Pichia) stipitis* was also reported (Agbogbo and Wenger 2007) during fermentation of corn stover hydrolysate. The product formed from acetic acid metabolism by *S. stipitis* CBS6054 is unknown. HMF and furfural are produced during the processing of hydrolysate, by degradation of hexose and pentose sugars, respectively. Apparently, the tested concentration levels of HMF and furfural were not affecting the fermentation and growth of *S. stipitis* CBS6054. Yeasts including *S. stipitis* can metabolize furfural to furfuryl alcohol and the enzyme NADH- dependent yeast alcohol dehydrogenase (ADH) is responsible for the reduction (Huang et al. 2009). In the present investigation, HMF and furfural were completely metabolized by the strain before significant utilization of sugars started. This was also previously reported by others (Almeida et al. 2008; Wan et al. 2012) and indicates that *S. stipitis* CBS6054 is readily capable of converting HMF and furfural in the tested lignocellulose hydrolysate from sugarcane bagasse. Cell growth was highest (0.079 g/l/h) in hydrolysate containing mixed sugars and inhibitors such as acetate, HMF and furfural at concentrations of 3.2 ± 0.1, 0.4 and 0.5 g/l, respectively, indicating that the processing of bagasse hydrolysate under this condition will not inhibit the growth of *S. stipitis*.

A lag phase of 12 hours is observed in the fermentation of C_GX_ hydrolysate. This lag phase is possibly due to a higher concentration of inhibitor in hydrolysate C_GX_ such as acetate (6.9 ± 0.1 g/l), HMF (1.2 g/l) and furfural (0.8 g/l). Similar observation was also reported by others (Agbogbo and Wenger 2007; Sreenath and Jeffries 2000). Although *S. stipitis* exhibited prolonged fermentation time for the hydrolysate processed at 200°C with 6 bar O_2_ containing the inhibitors at higher concentration, ethanol concentration up to 18.7 ± 1.1 g/l was obtained with an ethanol yield of 0.38 ± 0.02 g/g after 82 h. However, after adaptation to the hydrolysate C_GX_ within 12 h, exponential growth was observed. The performance was significantly improved shortly after 12 h of incubation. This lag phase can be overcome in a continuous process using initial high cell density and also by recycling the cells adapted to the inhibitors (Bellido et al. 2011).

## Competing interests

The authors declare that they have no competing interests.
